# Biology of Aging: Animal and Human Models

**DOI:** 10.1093/geroni/igaf122.1848

**Published:** 2025-12-31

## Abstract

Research addressing biology of aging and disease through model and human models will be presented.

